# Ion-Selective Carbon Nanotube Field-Effect Transistors for Monitoring Drug Effects on Nicotinic Acetylcholine Receptor Activation in Live Cells

**DOI:** 10.3390/s20133680

**Published:** 2020-06-30

**Authors:** Youngtak Cho, Viet Anh Pham Ba, Jin-Young Jeong, Yoonji Choi, Seunghun Hong

**Affiliations:** 1 Department of Physics and Astronomy and Institute of Applied Physics, Seoul National University, Seoul 08826, Korea; cyt920130@snu.ac.kr (Y.C.); pbvanh@hunre.edu.vn (V.A.P.B.); itsjjy@gmail.com (J.-Y.J.); yoonji0122@snu.ac.kr (Y.C.); 2 Department of Environmental Toxicology and Monitoring, Hanoi University of Natural Resources and Environment, Hanoi 11916, Vietnam

**Keywords:** carbon nanotube field-effect transistor, ion-selective membrane, valinomycin, nicotinic acetylcholine receptor, ion channel

## Abstract

We developed ion-selective field-effect transistor (FET) sensors with floating electrodes for the monitoring of the potassium ion release by the stimulation of nicotinic acetylcholine receptors (nAChRs) on PC12 cells. Here, ion-selective valinomycin-polyvinyl chloride (PVC) membranes were coated on the floating electrode-based carbon nanotube (CNT) FETs to build the sensors. The sensors could selectively measure potassium ions with a minimum detection limit of 1 nM. We utilized the sensor for the real-time monitoring of the potassium ion released from a live cell stimulated by nicotine. Notably, this method also allowed us to quantitatively monitor the cell responses by agonists and antagonists of nAChRs. These results suggest that our ion-selective CNT-FET sensor has potential uses in biological and medical researches such as the monitoring of ion-channel activity and the screening of drugs.

## 1. Introduction

Ionotropic receptors, or ligand-gated ion channels, are transmembrane proteins that allow ions to pass through the channel pore in response to the binding of specific chemicals. The receptors play many prominent roles, such as signal transmission in nervous systems and muscle contraction control [1,2,3,4]. For instance, the dysfunction of nicotinic acetylcholine receptors (nAChRs), ionotropic receptors that respond to acetylcholine and nicotine, can result in brain disorders such as Parkinson’s disease, schizophrenia, epilepsy, and neuromuscular junction disease [5,6,7,8,9,10]. Therefore, the monitoring of ionotropic receptors has been an important subject of biomedical research.

Conventional methods for the monitoring of receptor activities include radioactive binding assays, luminescence methods, and electrophysiological techniques [11,12,13,14,15]. However, these techniques have their own limitations. For example, binding assays require a time-consuming preparatory procedure, and some optical methods such as surface plasmon resonance can be utilized only for isolated receptor proteins or enzymes, not for whole cells. The patch-clamp technique is regarded as the gold standard for assessing ion channel functionality in live cells, but it requires complex equipment and a skilled operator [16,17]. On the other hand, field-effect transistor (FET) biosensors have been utilized for monitoring the activities of versatile biomolecules, exhibiting some advantages such as a rather straightforward operation and label-free detection [18,19,20,21,22,23,24,25,26,27,28]. Recently, FET-based biosensors have been studied as a tool to measure the electrical activities of live cells [29,30,31,32,33,34]. However, previous FET-based ion sensors often suffer from rather low sensitivity and can be used to monitor ion channel activities only in close proximity of individual cells. For this reason, the measurement of numerous cells for statistical analysis is often a challenging task.

Herein, we developed an ion-selective sensor based on a carbon nanotube field-effect transistor (CNT-FET) with floating electrodes for the quantitative monitoring of drug effects on nicotinic acetylcholine receptors (nAChRs) in live cells. In this study, a potassium ion-selective membrane was coated on a sensitive floating electrode-based CNT-FET so that potassium ions passing through the membrane could be detected by the underlying CNT-FET device. This sensor employs the advantages of FET sensors, such as label-free detection, fast response, and simple operational procedure. The floating electrodes enhanced the sensor sensitivity by the modulation of Schottky contact between carbon nanotube channels and the electrodes. Therefore, our sensors could selectively detect potassium ions at low concentrations down to 1 nM with a rather short response time of a few seconds. We utilized the sensor for the real-time monitoring of potassium ion release from a single PC12 cell stimulated by nicotine. Furthermore, the effects of various nAChR antagonists such as hexamethonium and mecamylamine on the cells were quantitatively measured using the sensor. This method allows one to selectively measure a specific ion and to monitor the ion channel activities of live cells with high sensitivity and selectivity. Thus, it can be a powerful tool for basic biological research and various clinical applications.

## 2. Materials and Methods

### 2.1. Materials

Semiconducting single-walled carbon nanotubes (s-SWCNTs) at 99% purity were purchased from NanoIntegris and used as received. The PC12 cell line was obtained from Korean Cell Line Bank (KCLB, Seoul, Korea) and supplies for cell culture such as RPMI 1640 medium, Hank’s Balanced Salt Solution (HBSS), phosphate-buffered saline (PBS), heat-inactivated horse serum, heat-inactivated fetal bovine serum, and penicillin-streptomycin were purchased from Gibco (Grand Island, NY, USA). Other chemical reagents including octadecyltrichlorosilane (OTS), valinomycin, potassium tetrakis(4-chlorophenyl) borate (KTClPB), bis(2-ethylhexyl) sebacate (dioctyl sebacate, DOS), polyvinyl chloride (PVC), tetrahydrofuran, 4-(2-hydroxyehyl)piperazine-1-ethanesulfonic acid (HEPES), N-methyl-D-glucamine (NMG), nicotine, acetylcholine chloride, hexamethonium bromide, mecamylamine hydrochloride, potassium chloride, sodium chloride, and lithium chloride were purchased from Sigma-Aldrich (St. Louis, MO, USA). Fluo-4 AM was purchased from Invitrogen (Waltham, MA, USA).

### 2.2. CNT-FET Preparation

**Figure 1:**
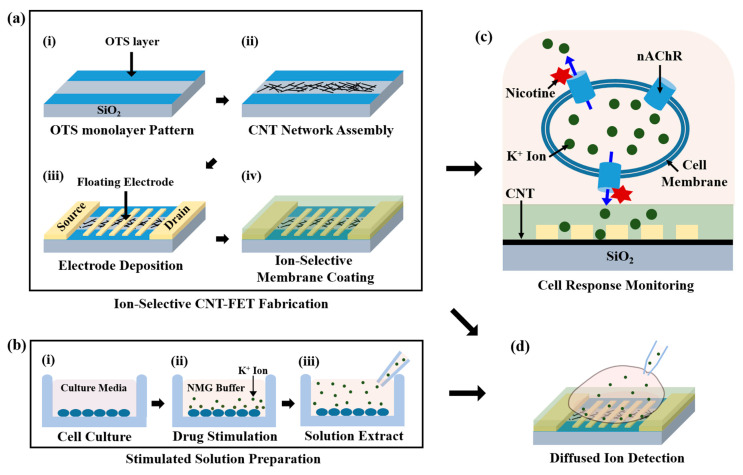
Schematic diagram depicting the procedures to prepare sensors and measure the potassium ions released from live cells. (**a**) Fabrication of an ion-selective carbon nanotube field-effect transistor (CNT-FET): (i) Patterning of octadecyltrichlorosilane (OTS) layers; (ii) specific adhesion of CNTs; (iii) deposition of floating electrodes on the CNT channel; (iv) coating of ion-selective membrane. (**b**) Cell preparation and stimulation: (i) culturing PC12 cells in an RPMI 1640 medium; (ii) stimulation of cells after media change; (iii) extraction of stimulated solution using a micropipette. (**c**) Direct monitoring of individual cell responses to nicotine using an ion-selective sensor. (**d**) Detection of potassium ion in the extracted solution using the sensor. The above drawing is not to scale.

Figure 1a illustrates the preparation of an ion-selective CNT-FET with floating electrodes. First, an OTS self-assembled monolayer with non-polar terminal groups was patterned onto a SiO_2_ substrate (300 nm) as reported previously (Figure 1a(i)) [35]. The substrate was exposed to the 1, 2-dichlorobenzene (DCB) solution of s-SWCNTs (0.025 mg/mL) for 3 min and thoroughly rinsed with pure DCB. As a result, the CNTs were selectively adsorbed onto the bare SiO_2_ surface without OTS monolayer and formed the network channel (Figure 1a(ii)) [35]. By using photolithography and thermal evaporation, metal electrodes (Pd/Au 10 nm/15 nm) were deposited on the CNT channel (Figure 1a(iii)). The source and drain electrodes were passivated with a photoresist (DNR-L300) layer to eliminate the leakage current during the electrical measurement in aqueous environments. The ion-selective membrane was prepared as reported previously [36]. In brief, the membrane ingredients were comprised of 2.8% valinomycin, 1.1% KTClPB, and 65% dioctyl sebacate in PVC. A total of 100 mg of components were dissolved in 1 mL of tetrahydrofuran, and 10 μL of the solution were dropped on the channel. Subsequently, the PVC-based membrane was formed by spin-coating (3000 rpm, 30 s) and drying under room temperature for 10 min (Figure 1a(iv)).

### 2.3. Preparation and Stimulation of PC12 Cells

The PC12 cell line derived from rat pheochromocytoma (KCLB no. 21721) was obtained from the Korean Cell Line Bank. The RPMI 1640 medium supplemented with 10% horse serum, 5% fetal bovine serum, and 1% penicillin-streptomycin (10,000 U/mL) was used as the culture medium. The cells were cultured in a humidified incubator with a 5% CO_2_ atmosphere at 37 °C (Figure 1b(i)). Before chemical stimulations, the cells were gently washed three times and incubated with an NMG buffer solution (pH 7.3, 135 mM NMG, 10 mM HEPES, 5 mM glucose, 2.2 mM CaCl_2_, and 1 mM MgCl_2_) for 1 h with a population density of 2.9 × 10^5^ cells/mL. Drug solutions, such as nicotine, were also prepared in the NMG buffer and then introduced to the cell media to stimulate the cells (Figure 1b(ii)). Drug-stimulated cell media were obtained using a micropipette at 5 min after the addition of drugs (Figure 1b(iii)). 

### 2.4. Electrical Measurements of Multiple Cells

A CNT-FET sensor was connected to a semiconductor analyzer (4200-SCS, Keithley), and a constant bias voltage of 0.1 V was applied between the source and drain electrodes during the electrical measurements. To provide an aqueous environment, 10 uL of NMG buffer solution were placed on the channel region of the FET. The sample solutions obtained from the stimulated cell media were added to the buffer solution, while a source-drain current was monitored (Figure 1c). In each addition, the applied solution had a volume of one-ninth of the solution above the channel region. Therefore, the molar concentrations of measured solutions were diluted to one-tenth of the sample solutions. In our experiments, the relative change of conductance ΔG/G_0_ was obtained by dividing the conductance change by the original conductance and used as a sensor signal. 

### 2.5. Statistical Analysis 

For the statistical analysis of sensor responses, the sensing measurements were repeated using at least three different sensor devices. Then, the means and standard errors of the means (S.E.M.) of the measured ΔG/G_0_ were calculated and utilized to represent data points and error bars on the graphs, respectively. For theoretical curve fitting for measured data, non-linear curve fittings were performed on OriginPro 8.0 software (OriginLab, Northampton, MA, USA) based on an iterative damped least-squares method. 

### 2.6. Fluorescence Imaging of Stimulated Cells

The PC12 cells were prepared by incubation on a 35 mm culture dish for more than one day. The medium was replaced from the culture medium to calcium-free HBSS before the experiment. Then, the cells were loaded with Fluo-4 AM at a final concentration of 5 μg/mL in the dark for 1 h at 37 °C. The culture dish was gently washed with the HBSS buffer to remove excess fluorescent dyes. The 300 μL of PBS solution containing 30 mM Ca^2+^ and 50 mM nicotine were introduced into 2.7 mL of the HBSS cell media to stimulate the cells. The fluorescence images of the cells were obtained using a fluorescence microscope (TE2000-U, Nikon, Tokyo, Japan) with an electron-multiplying charge-coupled device (CCD) camera (DQC-FS, Nikon, Tokyo, Japan), and a fluorescence excitation system (precisExcite, CoolLed, Andover, UK) at an excitation wavelength of 490 nm which is close to the excitation wavelength of ~488 nm for Fluo-4 AM dye. 

## 3. Results and Discussion

### 3.1. Characteristics of an Ion-Selective CNT-FET Sensor

**Figure 2:**
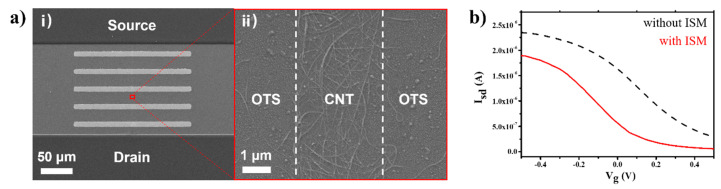
Characterization of an ion-selective CNT-FET with five floating electrodes. (**a**) Scanning electron microscope (SEM) images of (i) the whole channel region and (ii) the boundary of the CNT network region. (**b**) Source-drain current (I_sd_) versus gate voltage (V_g_) characteristics of a CNT-FET with (black) and without (red) ion-selective membrane (ISM). The CNT-FET showed p-type profiles in both cases.

Figure 2a shows the scanning electron microscope (SEM) images of a fabricated CNT-FET channel with floating electrodes. The SEM images were obtained by using an electron microscope (JSM-7800F Prime, JEOL) with the magnifications of 300× (Figure 2a(i)) and 15,000× (Figure 2a(ii)). Figure 2a(i) displays five floating electrodes between the source and drain electrodes with 10 μm × 200 μm dimensions for each electrode. A high-resolution SEM image shows the CNTs adsorbed on the 3 μm width area (Figure 2a(ii)). Note that the CNTs were selectively adsorbed on the bare SiO_2_ surface and formed a narrow channel, whereas OTS self-assembled monolayer blocked the adhesion of CNTs owing to their non-polar terminal groups [37,38]. These results verify that the CNT network channel and the floating electrodes of the device were successfully fabricated.

Figure 2b shows the gate profiles of a CNT-FET with and without the ion-selective PVC membrane. Here, the liquid gate bias (V_g_) was applied to deionized water on the channel region of the sensor using an Ag/AgCl electrode. The gate bias was swept from −0.5 V to 0.5 V, and source-drain currents (I_sd_) were measured while a source-drain bias was maintained at 0.1 V. The electric currents decreased with increasing gate voltage, which indicates a typical p-type characteristic of the CNT-FET. After the coating of the ion-selective membrane, the overall current levels were reduced. However, the p-type characteristics with a rather large transconductance under the external bias was maintained, indicating that the CNT-FET can be utilized as a highly-sensitive sensor transducer even with the membrane coating. 

### 3.2. Detection of Potassium Ion by Using Ion Sensor

**Figure 3:**
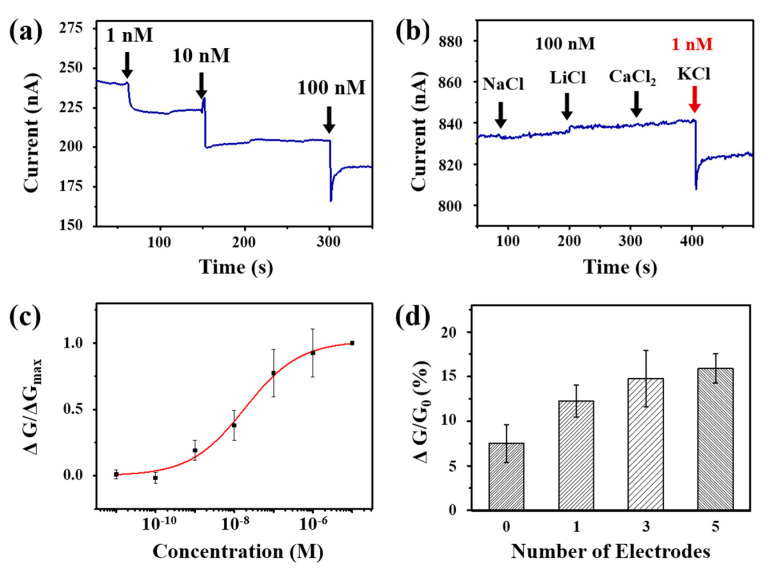
Responses of ion-selective CNT-FET sensors to the potassium ion solutions. (**a**) Real-time electrical current measurement of an ion-selective sensor during the addition of potassium ion solutions at different concentrations. The addition of solutions caused the decrease of electrical currents. (**b**) Real-time responses of a sensor to different ions from chloride solutions. The black and red arrows are representing additions of ion solution with concentrations of 100 nM and 1 nM, respectively. (**c**) Dose-dependent normalized conductance changes of potassium ions. (**d**) Relative conductance change of sensors with different numbers of floating electrodes to 10 nM potassium ion solution. Data are expressed as means ± standard errors of the means (S.E.M.) (*n* = 3).

Figure 3a shows the real-time measurement result of a source-drain current in a CNT-FET sensor during the addition of potassium ion solutions. Here, the source-drain voltage was maintained at 0.1 V during the measurement. The graph shows immediate decreases in the current after the addition of potassium solutions. Note that the sensor began to exhibit a significant current change when exposed to potassium ion at 1 nM concentration, indicating a better detection limit than previously-reported FET-based potassium sensors [39]. Furthermore, it is approximately eight orders of magnitude lower than intracellular potassium concentrations. This implies that our sensors have a sensitivity enough to monitor the potassium ions released from cells. A plausible explanation for the sensing mechanism can be the gating effect by the potassium ions transmitted through the ion-selective membrane. When the potassium solution was introduced, valinomycin within the ion-selective membrane formed K^+^-valinomycin complexes and transported the ions through the membrane as reported previously [40,41,42,43,44]. The K^+^ ions, which were transported to the CNT channel, provided a positive gate bias, resulting in a decrease of channel conductance due to the p-type characteristics of our CNT-FET devices, as shown in Figure 2b. We also confirmed that a bare CNT-FET without the membrane did not respond to the potassium ion solution (Figure S1). 

Figure 3b shows the real-time responses of an ion-selective sensor to various chloride solutions with different cations such as NaCl, LiCl, CaCl_2_, and KCl. Here, a source-drain current was monitored while introducing the solutions to the channel region with a source-drain bias of 0.1 V. Note that the addition of the ion solutions without K^+^ ions did not change the current levels. In contrast, the solution with two orders lower concentration of KCl caused a significant current decrease, indicating the high selectivity of our sensor. Presumably, the valinomycin in the membrane of our sensor can selectively form complexes only with potassium ions and provide the gating effects to the underlying CNT-FET sensor.

Figure 3c shows the dose-dependent curve of our sensors exposed to potassium ion solutions with different concentrations. The normalized signals were obtained by the normalization of the response signals with respect to their maximum signal values at high concentrations. We performed the measurements three times with different devices. Our sensors can detect potassium ions from the concentration of 1 nM and exhibited increased signals with increasing concentrations of potassium. Previous works show that the responses of common FET-based sensors can be explained by the Langmuir isotherm model with the Hill equation

(1) $$\Delta G/\Delta G_{\max}=\gamma \;\frac{C^{n}}{K_{D}^{\;n}{+C}^{n}}$$

where A is a conversion parameter, and n is the Hill coefficient. C is the concentration of potassium in a solution, and K_D_ is the dissociation constant of the potassium-valinomycin complex in the membrane [45,46]. The dissociation constant K_D_ and the Hill coefficient n were determined as 1.86 × 10^−9^ M and 0.62 by fitting the data to Equation (1), respectively. These values are similar to those reported in the literature [47,48,49]. 

To confirm the effect of floating electrodes, we fabricated the sensors with different numbers of floating electrodes and performed the sensing measurements (Figure S2). Figure 3d shows the sensor signals at the addition of 10 nM potassium ion solution. Note that the sensors with more electrodes exhibited larger sensor signals, indicating that the increased number of floating electrodes could result in the enhanced sensitivity of the CNT-FET sensors. This enhancement could be explained in terms of Schottky barrier modulation, one of the common sensing mechanisms for CNT-FET sensors (Figure S3) [50]. When floating electrodes are fabricated on a CNT channel, Schottky barriers are formed at the interfaces between the semiconducting CNTs and the metal electrodes. Here, the height of the Schottky barrier is affected by the work function of the electrodes. Thus, the binding of target molecules, which changes the work function of electrodes, can alter the height of the Schottky barrier. As a result, the channel conductance of the sensor is changed, enabling the sensing of target molecules. The previous report shows that the increased number of Schottky barriers is the main factor determining the enhancement of sensor sensitivity, while the shape or area of floating electrodes did not affect the sensitivity much [35]. Our results show that, with an increasing number of floating electrodes, the additional Schottky barriers caused the enhanced change in the channel conductance, as reported previously [35,51]. 

### 3.3. Activation of Ion Channel-Linked Receptor

**Figure 4:**
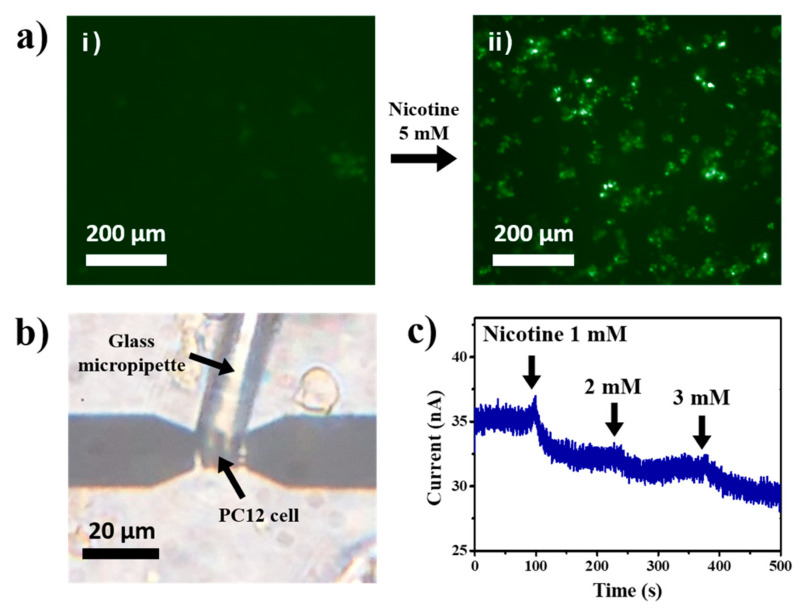
Real-time monitoring of ion-channel activation by nicotine in live cells. (**a**) Fluorescence images of PC12 cells (i) before and (ii) after the stimulation using 5 mM nicotine. The fluorescence occurred after the stimulation. (**b**) Optical image showing a PC12 cell placed on our sensor surface by a glass micropipette, for the electrical monitoring of the cell responses by the stimulation of nicotine. (**c**) Real-time electrical current change of a sensor with PC12 cells during the addition of nicotine solutions as shown in (**b**). The decrease in current was observed after the additions.

To confirm the expression of nicotinic receptors on PC12 cells, the activation of ion channel-linked receptors in the cells was monitored using a fluorescence method (Figure 4a). The PC12 cells were derived from the rat adrenal pheochromocytoma and retained some neuroendocrine characteristics such as the expression of nicotinic acetylcholine receptors (nAChRs) [52,53,54]. The detailed procedure of the fluorescence assay to monitor the effect of nicotine on the cells is described in Section 2.5. In brief, the cells were first incubated in a calcium-free buffer solution including Fluo-4 AM as a Ca^2+^ indicator so that the indicator diffused into the cells. Binding of nicotine to nAChRs resulted in the opening of the ligand-gated ion channels. Thus, the extracellular calcium ion flowed into the cell through the opened channels, and the Ca^2+^ indicator exhibited the fluorescence signal. Figure 4a shows the fluorescence images of PC12 cells before and after the addition of 5 mM nicotine. The cells showed a fluorescence signal after the addition of the nicotine solution. These results indicate that functional nAChR proteins were expressed in the membrane of the PC12 cells. As a control experiment, we performed the same fluorescence assay by adding only a buffer solution without nicotine (Figure S4). The results show that the change in fluorescence signals without nicotine was negligible, showing that the amount of calcium ions diffused through cell membranes was not significantand did not affectour measurements.

Figure 4b is the optical image of the PC12 cell placed on the ion-selective CNT sensor channel. Here, a cultured PC12 cell in sodium-free NMG buffer was picked up and placed on the channel regions of the sensor using a micromanipulator. Then, nicotine solutions were introduced while monitoring the source-drain current of the sensor. This method allows us to repeatedly measure the responses of individual cells using a single sensor device [23].

Figure 4c shows the real-time monitoring of a single PC12 cell response using our CNT-FET with the ion-selective membrane. The source-drain current was measured during the introduction of the nicotine solutions with different concentrations while maintaining the source-drain bias of 0.1 V. Note that the currents decreased immediately after the additions of nicotine, indicating the release of potassium ions from cells induced by nicotine. It also should be mentioned that the measured data show stable current signals for almost a hundred seconds before the addition of nicotine. It implies that the potassium ions diffused from cells without nicotine were negligible and did not affect our results. When the nAChRs were activated by ligands such as nicotine, the ion channels were opened and allowed the permeation of small monovalent and divalent cations through the pore of the channels [55,56]. On the other hand, the control experimental results show that the sensor without cells did not show any responses to the nicotine solution (Figure S5). It indicates that, in Figure 4c, the response of our sensor was caused by nicotine-induced cell activities. When the ion channels are opened, the ion concentration gradients across the cell membrane cause the influx of extracellular calcium and sodium ions and the efflux of intracellular potassium ion in the physiological condition of the mammalian cells. After that, the potassium ions diffused into a valinomycin-based membrane. The positive charge of potassium ion induced the decrease of the channel conductance as described previously (Section 3.2). Thus, the conductance change of our sensor could be regarded as a consequence of potassium ion release through the activated ion channel of the cell. The result shows that our ion-selective sensor could be used to monitor ion channel activation of an individual living cell. 

### 3.4. Drug Effect Monitoring Using Ion-selective Sensors

**Figure 5:**
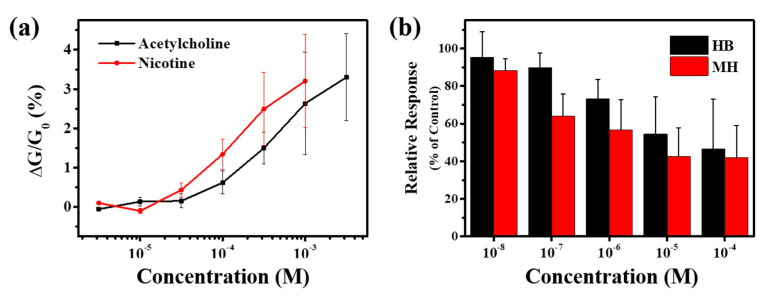
Quantitative monitoring of the effect of agonists and antagonists. (**a**) Conductance change with applied solutions stimulated by acetylcholine (black) and nicotine (red) at various concentrations. (**b**) Relative responses of solutions stimulated with hexamethonium (HB, black) and mecamylamine (MH, red). The inhibition effects of the drugs were observed. Data are expressed as means ± S.E.M. (*n* = 3).

For the statistical analysis of cell responses to drugs, we measured the drug responses of multiple live cells (Figure 5). Figure 5a shows the sensor responses to the medium solutions of the PC12 cells stimulated by different agonist drugs. Detailed experimental procedures were presented in Section 2.3. In brief, PC12 cells were first incubated in RPMI culture media for a day so that the cells could uptake potassium ions. Then, they were placed in the media without potassium and stimulated by a specific concentration of agonist. Previous works showed that agonist drugs such as nicotine and acetylcholine could activate the ion channels and induce the potassium outflow into the extracellular medium [11,53,57]. At five minutes after the addition of agonists, the sample solutions were obtained from cell media and used for the conductance change measurement of our sensors. For the measurement, the sample solution was introduced to the sensor channel, while the source-drain current of the sensor was monitored. The results show that our sensor exhibited increased responses to the sample solutions obtained from the cells stimulated with higher concentrations of agonists, indicating the cells released more potassium ions into the media (Figure 5a). By fitting the response data using the Hill equation, we could estimate the dissociation constant K_D_ of nicotine and acetylcholine to nAChRs as 3.05 × 10^–4^ ± 0.32 × 10^–4^ M and 8.70 × 10^–4^ ± 1.23 × 10^–4^ M, respectively. These results are similar to previously reported values, indicating that our sensor can be used to quantitatively monitor the ion-channel activity of PC12 cells induced by chemical stimuli [11,57,58].

The nAChRs have been reported to play a crucial role in the signal transmission of nervous systems and cell signaling pathways, which regulate various functions of organisms [55,56,57]. For instance, nicotine binding to ganglion-type nAChRs in the adrenal medulla increases heart rate and blood pressure by releasing adrenaline [59,60]. Moreover, chronic exposure to nicotine causes arterial stiffening and increases the prevalence of cardiovascular diseases, such as atherosclerosis and hypertension [61,62,63]. The inhibitors of nAChRs like hexamethonium and mecamylamine have been studied as drugs to treat chronic hypertension and addiction to nicotine [64,65,66,67,68]. They are non-competitive antagonists of nAChRs, which inhibit the activation of the receptor by not interfering with ligand binding. We measured the effect of antagonist drugs using our system. Figure 5b shows the measurement results of the quantitative inhibition effects of antagonists on receptor activation. In this experiment, PC12 cells were first pretreated with hexamethonium bromide (HB) or mecamylamine hydrochloride (MH) at different concentrations for five minutes. The pretreated PC12 cells were then stimulated by the addition of 5 mM nicotine, and the released potassium ions in the cell media were analyzed using our sensors. For statistical analysis, the measured sensor responses were normalized to that of the control group without inhibitor. Low concentrations of released potassium in the samples with the higher concentrations of antagonist drugs indicated that the nAChR activities were suppressed by HB (black) and MH (red) as reported previously [69,70]. The results were analyzed by the Hill equation for inhibitors

(2) $$\Delta G/G_{0}=\Delta G/G_{0,\;\max}+\left(\Delta G/G_{0,\;\min}-\Delta G/G_{0,\;\max}\right)\frac{A^{n}}{{{(\mathrm{IC}}_{50})}^{n}{+A}^{n}}$$

where A and IC_50_ are the concentration of an inhibitor and that of an inhibitor giving a half-maximal relative conductance change, respectively [71]. The potency of inhibitors could be evaluated by a pIC_50_ value which is defined as –log_10_(IC_50_) and represents an inhibition efficiency. By fitting the data, the pIC_50_ values for hexamethonium and mecamylamine were estimated to be 4.58 ± 0.85 and 5.74 ± 0.54, respectively. These values are consistent with previously-reported values [70,72,73]. These results clearly show that our method could be used to evaluate the effect of drugs on nAChR activation quantitatively. Considering that the quantitative measurements of drug effect by conventional methods are often laborious tasks with complicated procedures, our strategy can be considered as a useful strategy for practical applications such as drug screening and electrophysiological study of ion channel activities.

## 4. Conclusions

In summary, we successfully demonstrated a straightforward and potent method for the evaluation of drug effects on the activity of nAChR protein linked with ion channels. In this strategy, floating electrode-based CNT-FET sensors were modified by an ion-selective PVC membrane, enabling the quantitative detection of potassium ions with a high sensitivity. Our sensors with five floating electrodes could be used to detect potassium ions from the concentration of 1 nM and distinguish it from other ions. Using the sensor, we monitored the release of potassium ions from a single PC12 cell which was stimulated by nicotine in real-time. Moreover, the inhibition effect of channel-blocking drugs, hexamethonium and mecamylamine, was quantitatively measured in a concentration-dependent manner. This method could be a useful tool for various applications such as ionotropic receptor behavior study and drug screening.

## Supplementary Materials

The following are available online at https://www.mdpi.com/1424-8220/20/13/3680/s1, Figure S1: Real-Time Electrical Current Measurement of a CNT-FET without an Ion-selective Membrane during the Addition of Potassium Ions, Figure S2: Optical Images of CNT-FET Channels with Different Numbers of Floating Electrodes, Figure S3: Schematic Band Diagram of a Floating Electrode-Based CNT-FET, Figure S4: Fluorescence Image of PC12 Cells before and after the Addition of Buffer Solution without Nicotine, Figure S5: Real-Time Conductance Change of the Ion Sensor with and without Cell during the Addition of Nicotine.
