# Supplementary material for: Ion-Selective Carbon Nanotube Field-Effect Transistors for Monitoring Drug Effects on Nicotinic Acetylcholine Receptor Activation in Live Cells
Source: Sensors (Basel). 2020 Jun 30;20(13):3680. doi: 10.3390/s20133680 (PMC7374424; doi:10.3390/s20133680)
Supplement: Supplementary file 1 [file sensors-20-03680-s001.pdf]

[Supplementary Material]

Ion-Selective Carbon Nanotube Field-Effect Transistors for Monitoring Drug Effects on Nicotinic Acetylcholine Receptor Activation

Youngtak Cho[1], Viet Anh Pham Ba[1,2], Jin-Young Jeong[1], Yoonji Choi[1] and Seunghun Hong*[,1]

[1] Department of Physics and Astronomy and Institute of Applied Physics, Seoul National University, Seoul 08826, Korea

[2] Department of Environmental Toxicology and Monitoring, Hanoi University of Natural Resources and Environment, Hanoi 11916, Vietnam

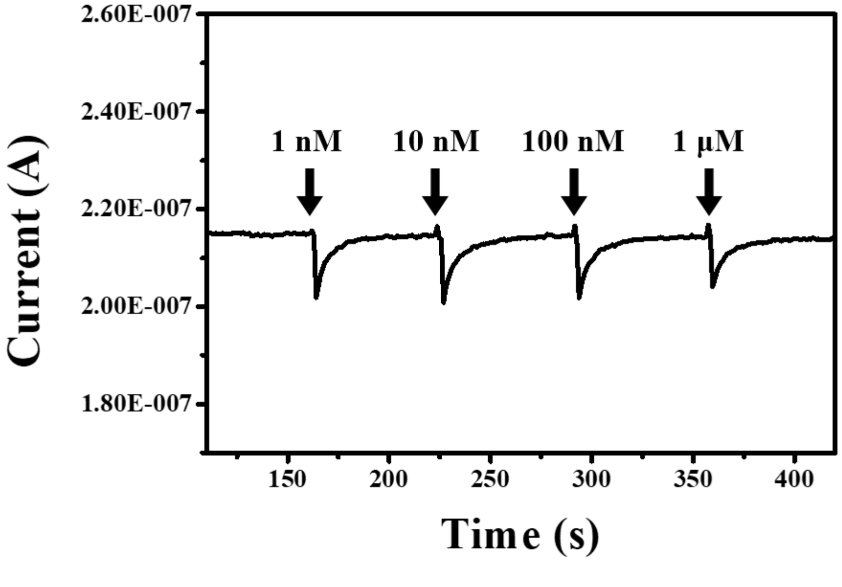

Figure S1. Real-Time Electrical Current Measurement of a CNT-FET without an Ion-selective Membrane during the Addition of Potassium Ions

Figure S1 shows the real-time measurement of a source-drain current in a CNT-FET without a potassium-selective membrane during the addition of KCl solutions with concentrations from 1 nM to 1 μM. Here, the source-drain voltage was maintained at 0.1 V during the measurement. The FET device did not show the decrease of current as the KCl solutions were applied. In the absence of the ion-selective membrane, there is no gate bias to differentiate the source-drain current, due to the formation of the electrical double layer on the CNTs.

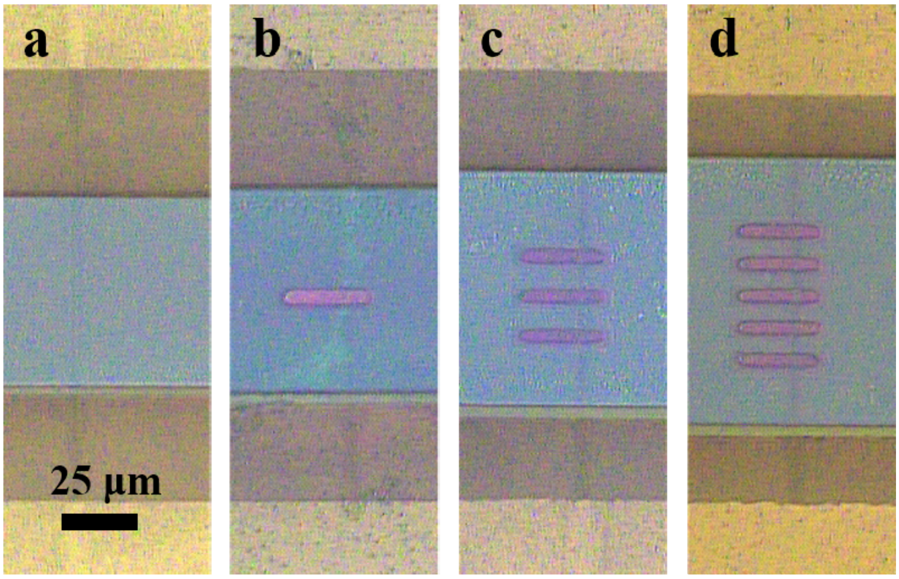

Figure S2. Optical Images of CNT-FET Channels with Different Numbers of Floating Electrodes

Figure S2 shows the optical images of CNT-FET channel region with (**a**) 0, (**b**) 1, (**c**) 3, and (**d**) 5 floating electrodes. The images were obtained by using an optical microscope (XY-MRT, Sunny) equipped with a charge-coupled device camera (SDC-415S, Samsung). A floating electrode has a width of 25 µm and a length of 4 µm. All devices had channels with exposed CNT network length of 50 µm. The length of the channels are (a) 50, (b) 54, (c) 62, and (d) 70 µm, and the distance between floating electrodes and source or drain electrodes are (b) 25, (c) 19, and (d) 17 µm. The CNT-FETs with identical CNT channel length and different numbers of floating electrodes were fabricated to verify the effect of floating electrodes.

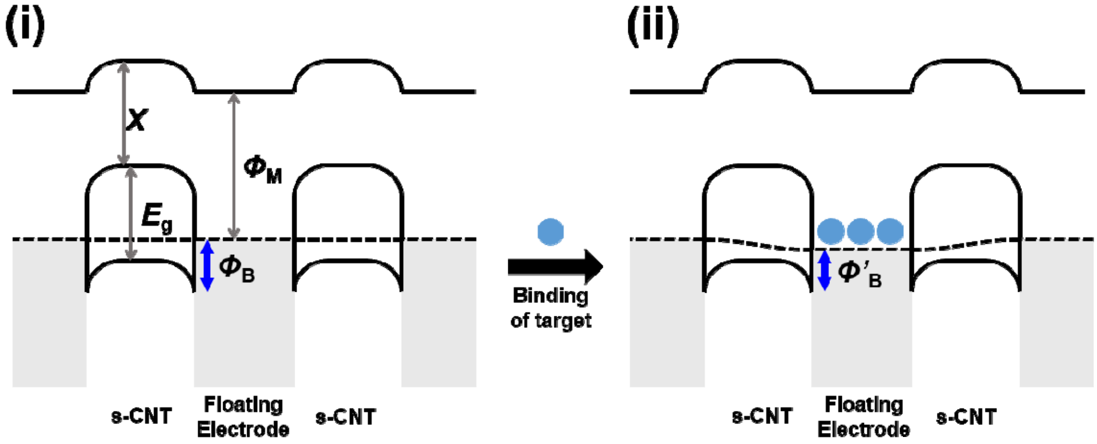

Figure S3. Schematic Band Diagram of a Floating Electrode-Based CNT-FET

Figure S3 shows the energy band diagram of a floating electrode-based CNT-FET sensor (i) before and (ii) after the binding of target molecules. Φ_B is the height of the Schottky barrier, and Φ_M is the work function of the floating metal electrode. X and E_g are an electron affinity and a band gap energy of semiconducting CNTs, respectively. As shown in the figure, the barrier height could be represented as $\Phi_B = E_g - (\Phi_M - X)$. Here, the binding of target molecules changes the work function of the floating electrode and, hence, modifies the height of the Schottky barrier, resulting in the conductance change of the CNT-FET.

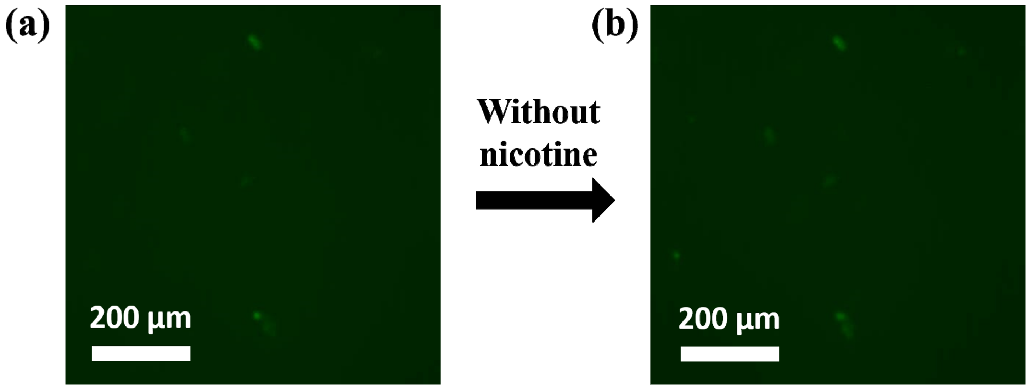

Figure S4. Fluorescence Image of PC12 Cells before and after the Addition of Buffer Solution without Nicotine

Figure S4 shows the fluorescence images of PC12 cells (**a**) before and (**b**) after the addition of a buffer solution without nicotine. In contrast to (a), we could not observe any significant change of fluorescence signals in the measurement. The results show that the diffused calcium ions through cell membranes without nicotine were negligible.

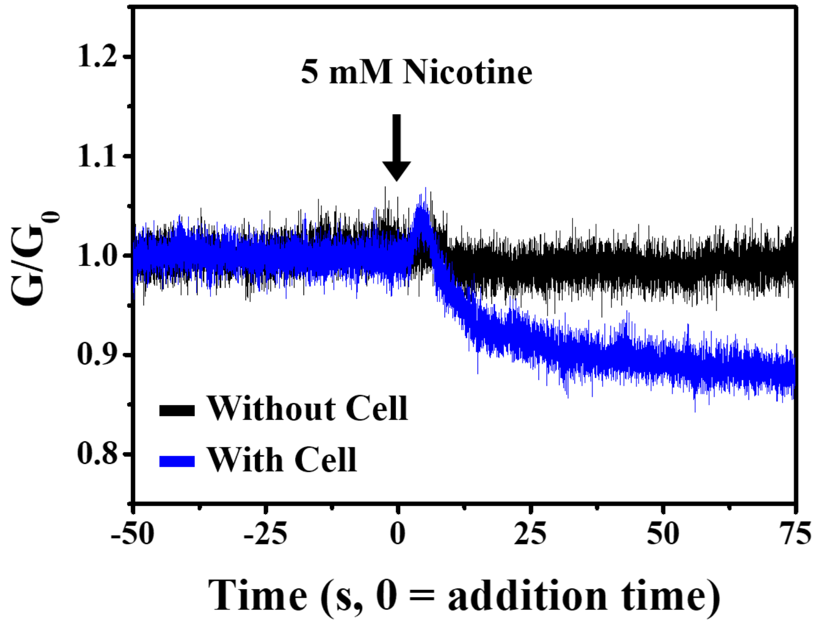

Figure S5. Real-Time Conductance Change of the Ion Sensor with and without Cell during the Addition of Nicotine.

Figure S5 shows the real-time conductance change of the ion-selective sensor during the addition of nicotine with and without PC12 cell on the channel. The source-drain voltage was maintained at 0.1 V during the measurement. The time point at the addition of nicotine was represented as 0 s. The graph shows an immediate decrease in the conductance after the addition of nicotine when the cell is on the CNT channel. Meanwhile, in the absence of the cell, the sensor did not show a significant change in the conductance. This result indicates that the response of the sensor was caused by the potassium released from the cell, not the nicotine itself.
